# Social Jetlag and Chronotypes in the Chinese Population: Analysis of Data Recorded by Wearable Devices

**DOI:** 10.2196/13482

**Published:** 2019-05-11

**Authors:** Zhongxing Zhang, Christian Cajochen, Ramin Khatami

**Affiliations:** 1 Center for Sleep Medicine, Sleep Research and Epileptology Clinic Barmelweid AG Barmelweid Switzerland; 2 Centre for Chronobiology Psychiatric Hospital of the University of Basel Basel Switzerland; 3 Transfaculty Research Platform Molecular and Cognitive Neurosciences University of Basel Basel Switzerland; 4 Department of Neurology Inselspital Bern University Hospital and University of Bern Bern Switzerland

**Keywords:** chronotypes, social jetlag, wearable devices, nap, cardiopulmonary coupling, sleep, big data

## Abstract

**Background:**

Chronotype is the propensity for a person to sleep at a particular time during 24 hours. It is largely regulated by the circadian clock but constrained by work obligations to a specific sleep schedule. The discrepancy between biological and social time can be described as social jetlag (SJL), which is highly prevalent in modern society and associated with health problems. SJL and chronotypes have been widely studied in Western countries but have never been described in China.

**Objective:**

We characterized the chronotypes and SJL in mainland China objectively by analyzing a database of Chinese sleep-wake pattern recorded by up-to-date wearable devices.

**Methods:**

We analyzed 71,176 anonymous Chinese people who were continuously recorded by wearable devices for at least one week between April and July in 2017. Chronotypes were assessed (N=49,573) by the adjusted mid-point of sleep on free days (MSFsc). Early, intermediate, and late chronotypes were defined by arbitrary cut-offs of MSFsc <3 hours, between 3-5 hours, and >5 hours. In all subjects, SJL was calculated as the difference between mid-points of sleep on free days and work days. The correlations between SJL and age/body mass index/MSFsc were assessed by Pearson correlation. Random forest was used to characterize which factors (ie, age, body mass index, sex, nocturnal and daytime sleep durations, and exercise) mostly contribute to SJL and MSFsc.

**Results:**

The mean total sleep duration of this Chinese sample is about 7 hours, with females sleeping on average 17 minutes longer than males. People taking longer naps sleep less during the night, but they have longer total 24-hour sleep durations. MSFsc follows a normal distribution, and the percentages of early, intermediate, and late chronotypes are approximately 26.76% (13,266/49,573), 58.59% (29,045/49,573), and 14.64% (7257/49,573). Adolescents are later types compared to adults. Age is the most important predictor of MSFsc suggested by our random forest model (relative feature importance: 0.772). No gender differences are found in chronotypes. We found that SJL follows a normal distribution and 17.07% (12,151/71,176) of Chinese have SJL longer than 1 hour. Nearly a third (22,442/71,176, 31.53%) of Chinese have SJL<0. The results showed that 53.72% (7127/13,266), 25.46% (7396/29,045), and 12.71% (922/7257) of the early, intermediate, and late chronotypes have SJL<0, respectively. SJL correlates with MSFsc (r=0.54, *P*<.001) but not with body mass index (r=0.004, *P*=.30). Random forest model suggests that age, nocturnal sleep, and daytime nap durations are the features contributing to SJL (their relative feature importance is 0.441, 0.349, and 0.204, respectively).

**Conclusions:**

Our data suggest a higher proportion of early compared to late chronotypes in Chinese. Chinese have less SJL than the results reported in European populations, and more than half of the early chronotypes have negative SJL. In the Chinese population, SJL is not associated with body mass index. People of later chronotypes and long sleepers suffer more from SJL.

## Introduction

Many human biological processes and behavioral functions exhibit 24-hour rhythms driven by endogenous circadian clocks, ranging from metabolism, hormone secretion, and body temperature to sleep-wake patterns and socialization. These endogenous daily variations among humans can be determined by “chronotype”, which is controlled by circadian clocks but can be influenced by external environmental factors. Social jetlag (SJL) is the misalignment between biological and social time [1]. It is highly prevalent in modern society because of different factors such as increased workload and work stress in daily life, shift work, environmental changes [2] (eg, climate changes [3]), increasing exposure to artificial light sources, as well as excessive tablet/smartphone use [4]. SJL is reported to be associated with many health problems like obesity [5], increased daytime sleepiness and fatigue [6,7], bad mood and depression [8], and metabolic and cardiovascular disorders [9]. Reducing SJL is an important issue in public health.

One cause of SJL is insufficient sleep during work days, when people need to wake up earlier than their normal biological time and subsequently oversleep on free days to compensate for the accumulated sleep debt during the week [1,5]. Therefore, it is reasonable to assume that regular daytime naps to counteract accumulating sleep debt would alleviate SJL symptoms. Currently, most studies on SJL have been performed in Western countries [10,11]. It is difficult to investigate the relationship between daytime naps and SJL in these countries, since typically the general public rarely takes naps. Although “siesta” is historically common in Mediterranean countries such as Spain and Italy, Spaniards and Italians are abandoning this tradition and the majority of people no longer take naps [12]. In contrast, the noon nap is a Chinese tradition and is well protected in modern China despite the country’s rapid industrialization and economic boom. Chinese people consider napping at noon as important for their health, and the noon nap is usually mandatory for kids at school. Chinese adults commonly take daily noon naps to refresh their energy for the activities in the afternoon and continue with this habit even after retirement [13]. Typically, Chinese people have a 2-hour break at noon that allows them to eat lunch and then take a nap.

To the best of our knowledge, the current states of SJL, chronotypes, and sleep in the Chinese population have never been quantified. Among several reasons for the lack of Chinese data is the absence of a Chinese translation and validation of questionnaires used for studying SJL and chronotypes, such as the Munich ChronoType Questionnaire (MCTQ) [14]. Some questionnaires evaluating chronotypes such as Morningness-Eveningness Questionnaire (MEQ) and its short form, the reduced MEQ, have been translated into Chinese [15,16] but lack sufficient validation. China is a huge country with 56 different ethnic groups, and for these groups, a countrywide validation has not yet been done. Certain studies [15,16] show inconsistent validation results of chronotypes in the Beijing region, which limits them from application in large population studies.

Wearable devices are becoming increasingly popular. Some have been validated for human sleep measurements in daily life situations, making it possible to assess sleep, chronotype, and SJL in the general population during daily life in an objective and longitudinal way. However, wearable devices measuring sleep based only on accelerometers overestimate sleep duration as they cannot really distinguish sleeping from lying quietly [17-21]. Recently, new wearables devices have been improved by adding the function of monitoring autonomic activities such as heart rate and pulse wave using photoplethysmography [22] or electrocardiogram sensors [23]. With the help of novel algorithms like machine learning [22,24] and cardiorespiratory sleep staging techniques (eg, cardiopulmonary coupling [CPC] [23,25] and heart rate variability analysis [26]), movement tracking (ie, accelerometer) and autonomic activities have improved the detection and determination of sleep and wakefulness [22,24,26]. Therefore, these new wearable devices are becoming more attractive in sleep and chronobiology research [23,27,28].

In this study, we characterize sleep, chronotypes, and SJL in mainland China by analyzing a large database of Chinese sleep-wake pattern recorded by up-to-date smartwatches. The device used in this study assesses the sleep-wake state by measuring movements and CPC with accelerometers and photoplethysmography. We hypothesize that the Chinese population has a smaller SJL compared to the data published in European countries, since napping at noon is very common in China. Given that insufficient sleep during the work day is one of the potential contributors to SJL [1,5], we reason that the Chinese “noon nap” culture is helpful for reducing daily sleep debt and counteracting SJL.

## Methods

We analyzed 71,176 anonymous Chinese customers (male: 45,582 [64.04%], female: 9714 [13.65%], unknown: 15,880 [22.31%]) who use a wearable device from a major Chinese technology and telecommunications brand. For privacy reasons, we cannot reveal the name of the company. All customers were continuously recorded by their smartwatches for at least one week between April and July in 2017. Their nocturnal sleep durations were between 3 hours and 13 hours, that is, people who slept less than 3 hours or more than 13 hours were excluded. The age of these subjects was between 10 and 90 years old. We excluded customers with extremely high (>50 kg/m^2^) or low (<10 kg/m^2^) body mass indices (BMI). All subjects provided an electronic informed consent for their data to be used for research purposes when they first registered and initialized their devices. Hence, the data were collected naturally and safely during the customers’ daily life. The wearable devices are able to distinguish sleep and wakefulness with high accuracy by combining movement tracking and CPC assessment. The accuracy of the wearable devices assessing sleep has been validated by comparing with in-lab video-polysomnography [25,29]. This study was approved by the scientific and executive board of Clinic Barmelweid, Switzerland.

Mid-sleep point is the middle time between sleep onset and waking; for example, mid-sleep point is 4:00 am if sleep onset is at 00:00 and wake up time is 8:00 am in the morning. In each subject, we first calculated the mid-sleep points of all nights and then averaged the values on work days (from Sunday night to Thursday night) and on free days (Friday and Saturday nights), respectively. SJL is calculated as the averaged mid-sleep points on free days minus the averaged mid-sleep points on work days.

Changes of the adjusted mid-point of sleep on free days (ie, mid-sleep on free days corrected for sleep debt on work days) (MSFsc) were also characterized in 49,573 subjects (male: 30,651 [61.83%], female: 6394 [12.90%], unknown: 12,528 [25.27%]) whose data included at least 2 whole weeks with no missing data for age. In this way, we could test if the relationship between MSFsc and age as reported in a previous study [30] can be reproduced in the Chinese population. In addition, MSFsc measures human chronotype. Assessing chronotype is relevant for SJL because people of later chronotypes (ie, more evening type) are more likely to have higher sleep debt on work days and consequently a higher SJL [5]. The MSFsc was calculated according to the literature [30] as follows:

*MSFsc*=
*MSF* –0.5*[
*SD*_f_–(5*
*SD*_w_+2*
*SD*_f_)/7]

where *SD*_f_ was the mean sleep duration of Friday and Saturday nights, and *SD*_w_ was the mean sleep duration from Sunday to Thursday nights. As these 49,573 subjects had recordings of whole weeks, MSFsc of each subject was the mean value of the MSFsc of recording weeks. For example, if a subject was recorded for 3 weeks, we calculated the MSFsc of each week and then the average of the MSFsc of these 3 weeks was the final MSFsc of this subject. Then we classified the chronotypes into early, intermediate, and late types with the arbitrary cut-offs (ie, early: <3:00 am intermediate: 3:00-5:00 am, late: >5:00 am) as suggested by Roenneber et al [31].

Three subgroups of subjects were defined according to the average durations of their daytime nap: (1) group with long naps, that is, average nap duration longer than 30 minutes (n=29,917), (2) group without daytime nap whose average nap duration was 0 (n=4223), and (3) group with short naps, that is, the rest of the subjects (n=37,036) taking daytime naps shorter than 30 minutes. A two-sample *t* test was used to check if SJL showed gender differences in all subjects and in the long naps and zero naps groups, respectively. The correlations between SJL and age/BMI/MSFsc were assessed by Pearson correlation in all subjects and in the long naps and zero naps groups, respectively. The short naps group was excluded from the aforementioned analysis because their mean nap duration was short and the nap effect may be minor in some subjects. Linear regression was used to quantify the relationship between SJL and daytime nap/nocturnal sleep durations. The data were expressed as mean ± standard deviation (SD), unless otherwise stated. The statistically significant level was *P*<.001, considering the large sample size.

We used random forest [32], a supervised machine learning approach that can be used for both classification and nonlinear regression analysis, to characterize which factors influence SJL and MSFsc. Random forest can handle highly nonlinear interactions between variables and does not require distributional assumptions (ie, normality) for the variables. The predictors included sex, age, BMI, the count of daily walking steps (an indicator of exercise), nocturnal sleep duration, and daytime nap duration. Although classical linear regression analysis was frequently used to estimate the relationships among variables, it was not suitable for our dataset due to the multicollinearity (ie, the covariates were highly correlated to each other). To eliminate the influences of missing data on the weights of the predictors, we fitted only the random forest model with complete dataset of all the predictors (n=32,020). The number of trees was set from 10 to 50 with a stepwise increment of 10, and the maximal depth of the tree was set from 2 to 5 with a stepwise increment of 1. We used a 5-folder cross-validation to select the optimal model, which gave the highest score (ie, the score was the coefficient of determination R-squared of the random forest regression). All the statistical analyses and random forest regression were done using Python.

## Results

### Sleep Duration

**Figure 1:**
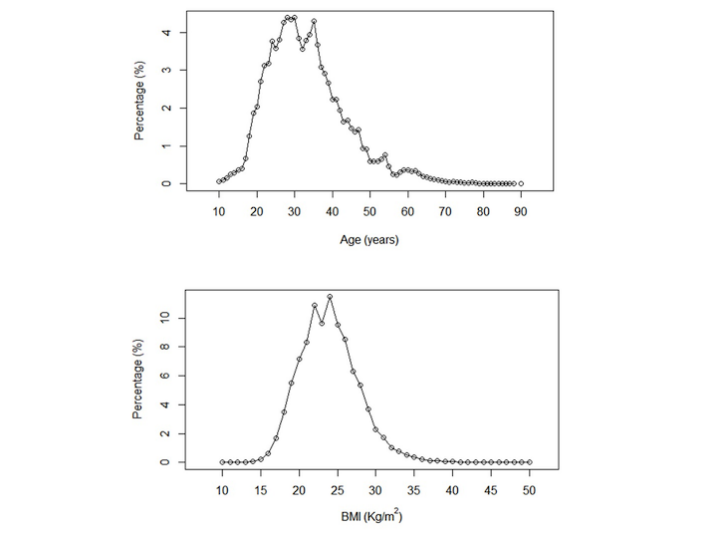
The distribution of age and body mass index (BMI) of our subjects.

**Figure 2:**
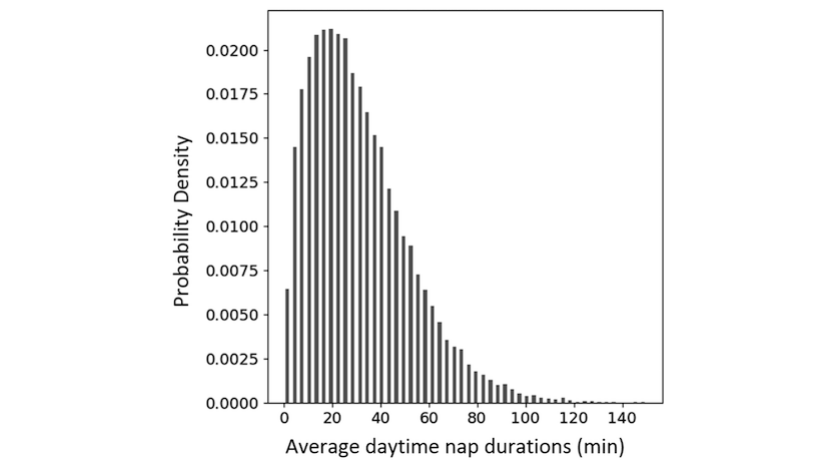
The distribution of the duration (>0 minutes) of daytime naps.

The distributions of age and BMI of our subjects are shown in Figure 1. The majority of our subjects were between 20 and 50 years old, with a BMI typically between 17 and 30 kg/m^2^. On average, subjects (n=71,176) fell asleep at 00:15 am (SD 70.5 minutes) and woke up at 7:00 am (SD 66.3 minutes) on work days, while they fell asleep at 00:28 am (SD 82 minutes) and woke up at 7:22 am (SD 81.3 minutes) on free days. Thus, their mean nocturnal sleep durations were 387 minutes (SD 49.5) on work days and 396.6 minutes (SD 65.8) on free days. Their nocturnal sleep was 10 minutes longer on free days compared to work days (Welch’s *t* test gives *P*<.001). The vast majority of our subjects took a daytime nap (66,953 out of 71,176 people, which was approximately 94.07% of our subjects). The distribution of the durations (>0 minutes) of the daytime naps is shown in Figure 2. Most of our subjects took naps shorter than 60 minutes. The total 24-hour sleep durations were 419 minutes (SD 47) (ie, nocturnal durations plus daytime nap durations), which were longer in females (433 minutes, SD 47) than males (416 minutes, SD 47) (two-sample *t* test, *P*<.001). We found a correlation between total sleep durations on work days and on free days in all subjects (*r*=0.41, *P*<.001), as well in the subgroups with zero naps (*r*=0.43, *P*<.001), with short naps (*r*=0.42, *P*<.001), and with long naps (*r*=0.37, *P*<.001), indicating that people who slept longer during work days also slept longer on weekends.

The nocturnal sleep duration negatively correlated with BMI (*r*=-0.216, *P*<.001) and with age (*r*=-0.14, *P*<.001). But no correlation was found between nap durations and BMI (*r*=0.003, *P*=.54). Further, nap durations did not correlate with age (*r*=0.05, *P*<.001), since the correlation coefficient was very small in spite of a small *P* value. It was thus not surprising to find negative correlations between total sleep durations and BMI (*r*=-0.21, *P*<.001) as well as between total sleep durations and age (*r*=-0.12, *P*<.001), since nocturnal sleep duration was much longer than nap duration.

**Figure 3:**
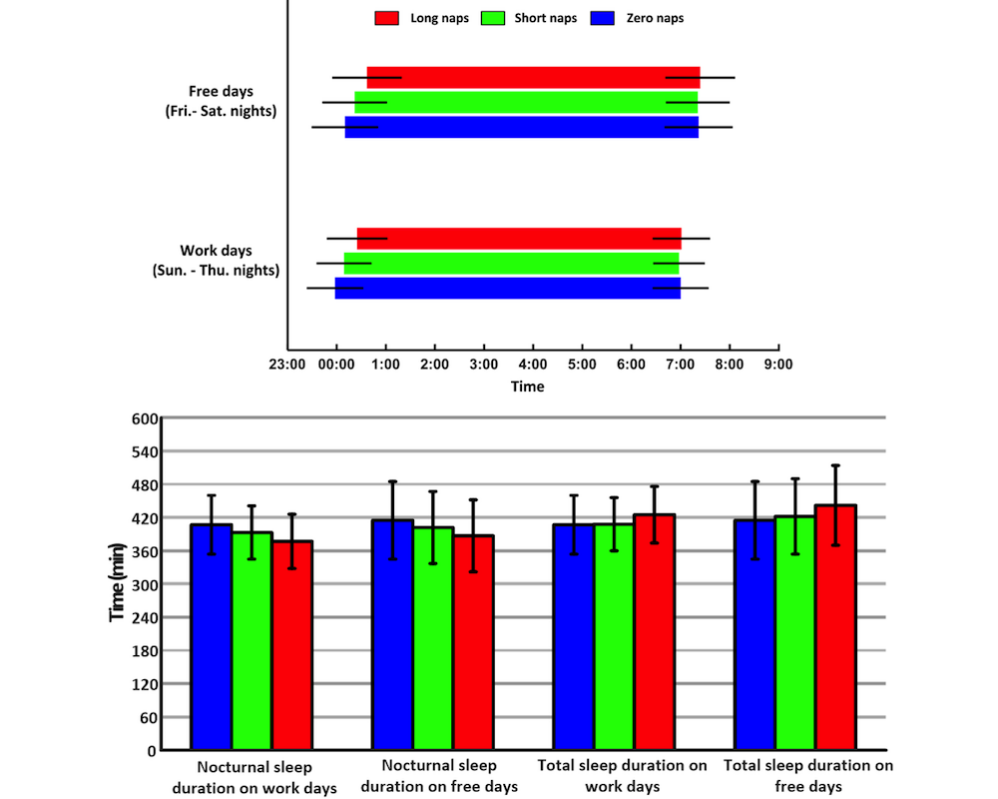
The sleep schedules and sleep durations on work and free days in the 3 subgroups with zero naps, short naps (≤30 minutes), and long naps (>30 minutes). The error bars are standard deviation. The group of zero naps has longer nocturnal sleep duration on both work (*P*<.001) and free days (*P*<.001) than the other two groups, and the group of short naps has longer nocturnal sleep on both work (*P*<.001) and free days (*P*<.001) than the long naps group. However, people taking longer daytime naps have significantly longer total sleep durations across the 24-hour day on both work (*P*<.001) and free days (*P*<.001) than the other two groups.

Figure 3 summarizes the sleep schedules of the three subgroups (ie, zero naps, short naps, and long naps). People taking longer daytime naps fell asleep later on both work and free days, but people in the three subgroups woke up at a similar time in the morning. The sleep schedules slightly shifted later on free days compared to work days in all the subgroups. After removing the durations of wake after sleep onset from sleep recordings, the average sleep durations of the three subgroups are illustrated in Figure 3. The group of zero naps had longer nocturnal sleep duration on both work and free days than the other two groups, but people taking longer daytime naps had significantly longer total sleep durations across the 24-hour day. This was confirmed by correlation analysis as nocturnal sleep duration was negatively correlated with nap duration (*r*=-0.22, *P*<.001).

**Mid-Sleep on Free Days Corrected for Sleep Debt on Work Days**

**Figure 4:**
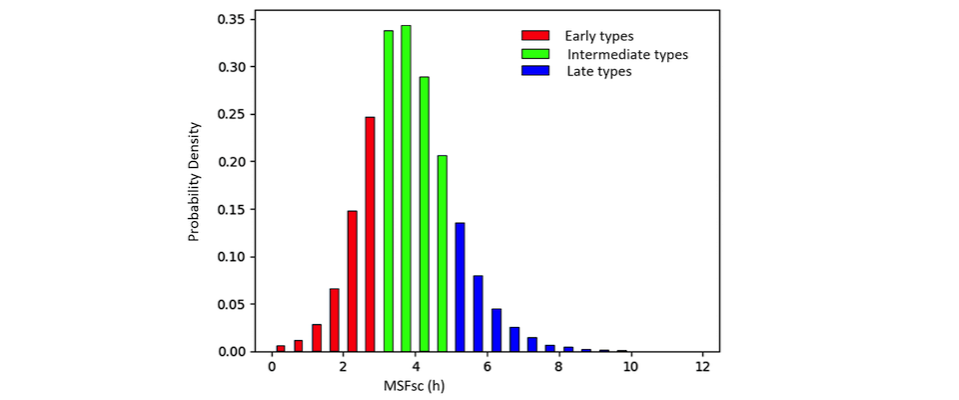
The distribution of adjusted mid-point of sleep on free days (MSFsc) in our subjects (N=49,573). The red, green, and blue colors mark the early, intermediate, and late types using arbitrary cut-offs of <3 am, 3-5 am, and >5 am, respectively. h: hours.

**Figure 5:**
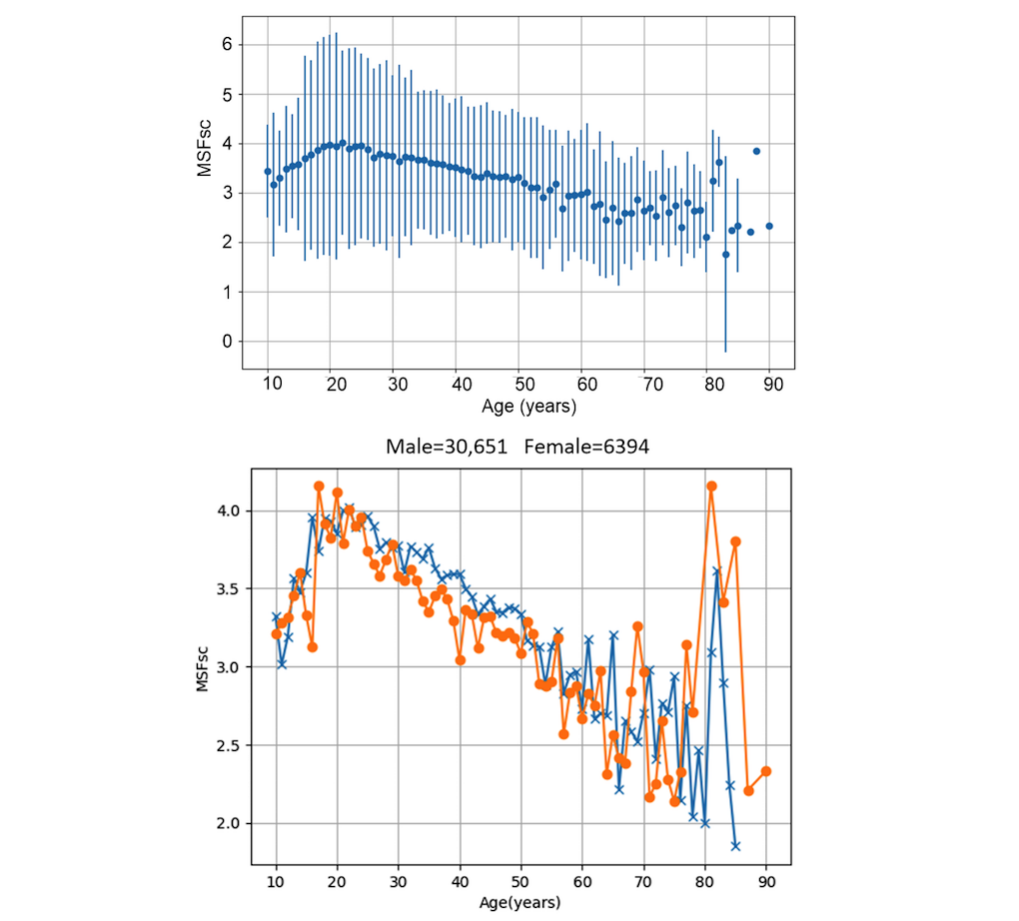
Age-dependent changes in adjusted mid-point of sleep on free days (MSFsc), and age-dependent changes of MSFsc in males and females (orange line with filled circles=female). The error bar is standard deviation.

The distribution of MSFsc is shown in Figure 4. We found a near-normal distribution of chronotypes, with the most frequent MSFsc between 3:00 am and 4:00 am. The percentages of early (red color in Figure 4), intermediate (green color in Figure 4), and late (blue color in Figure 4) types were approximately 26.76% (13,266/49,573), 58.59% (29,045/49,573), and 14.64% (7257/49,573) in our subjects. The trajectory of age-dependent changes in MSFsc (Figure 5) showed a biphasic pattern with the peak value of 4:00 am at the age of 22 years. The MSFsc at 22 years was significantly later than the ones at ages younger than 16 years and older than 26 years (Welch’s *t* test, *P*<.001). No gender differences were found (Welch’s *t* test, *P*>.05) in all age groups except for the ones of 34 years (*P*<.001), 35 years (*P*<.001), 39 years (*P*<.001), and 40 years (*P*<.001).

The optimal model of random forest regression was constructed with 50 trees with tree depth of 4. The relative feature importance for sex, age, BMI, exercise, nocturnal sleep duration, and daytime nap duration was 0.024, 0.772, 0.023, 0.036, 0.056, and 0.089, respectively, indicating that age was the predominant factor predicting MSFsc (ie, chronotype) in Chinese population. However, gender was not an important factor influencing MSFsc. The relative feature importance of nocturnal and daytime sleep durations suggested that they did not influence MSFsc in Chinese, as confirmed by correlation analyses. That is, nocturnal sleep (*r*=0.0048, *P*=.39) and nap durations (*r*=-0.008, *P*=.15) did not correlate to MSFsc.

### Social Jetlag

**Figure 6:**
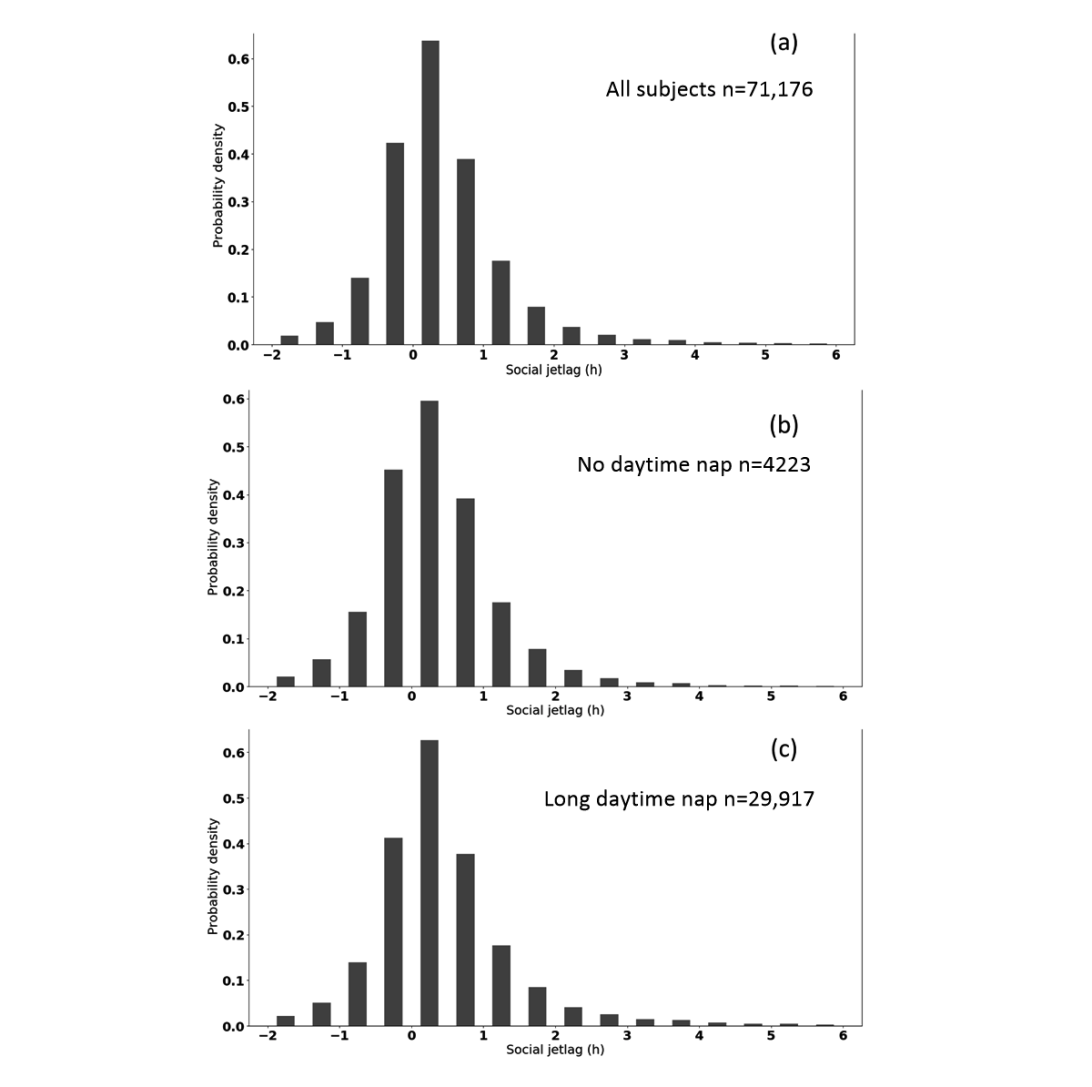
Histogram of social jetlag of (a) all subjects, (b) people with zero naps, and (c) people with long naps. h: hours.

Only 17.07% (12,151/71,176) of Chinese had SJL of more than 1 hour (see the distribution of SJL of our subjects in Figure 6). The distributions of SJL in the zero naps and long naps subgroups were similar to that of all the subjects. Figure 6 also shows that quite a number (22,442/71,176, 31.53%) of people had SJL<0. We further analyzed the number of negative SJL in different chronotypes (the chronotypes of 49,573 subjects were calculated as introduced above). We found 31.16% (15,447/49,573) of Chinese had negative SJL, that is, their mid-sleep points on work days were later than the ones on free days. Results showed that 46.14% (7127/15,447) of these subjects were early types, 47.88% (7396/15,447) intermediate types, and only 5.97% (922/15,447) late types. In other words, just over half (7127/13,266, 53.72%) of all the early types Chinese showed negative SJL. This proportion was 25.46% (7396/29,045) in the intermediate and 12.71% (922/7257) in the late types.

Social jetlag did not correlate with BMI (all subjects without missing values of BMI: n=58,370, *r*=0.004, *P*=.30; subgroup with zero naps: n=3324, *r*=0.017, *P*=.34; subgroup with long naps: n=24,891, *r*=-0.007, *P*=.30). When further dividing our subjects into two subgroups (ie, BMI≥25 and BMI<25), no correlation was found between SJL and BMI in either group (BMI≥25: n=23,841, *r*=-0.009, *P*=.36; BMI<25: n=34,529, *r*=-0.002, *P*=.78). Taken together, these results indicate that SJL may not be associated with increased BMI. No gender difference was found in SJL in all subjects and the two subgroups of zero and long naps (male vs female in all subjects: 0.29 hours [SD 1.25] vs 0.31 hours [SD=1.02], *P*=.06; in the zero naps group: 0.26 hours [SD 1.19] vs 0.25 hours [SD 0.85], *P*=.86; in the long naps group: 0.3 hours [SD 1.42] vs 0.3 hours [SD 1.15], *P*=.77). Social jetlag significantly correlated to MSFsc in all subjects (*r*=0.54, *P*<.001) and in the two subgroups with zero daytime naps (*r*=0.56, *P*<.001) and with long daytime naps (*r*=0.58, *P*<.001). This result suggests that SJL was related to chronotypes, that is, people of later chronotypes may suffer more from SJL.

As MSFsc significantly correlated with SJL, we excluded it from the predictors in our random forest regression as we were interested in finding other predictors of SJL. Random forest regression showed that the best model was built with 40 trees with a depth of 2. The relative feature importance for sex, age, BMI, exercise, nocturnal sleep duration, and daytime nap duration was 0, 0.441, 0.006, 0, 0.349, and 0.204, respectively, suggesting that within the available predictors, age was the most important contributor to SJL, followed by the durations of nocturnal sleep and daytime nap. These results also suggest that sex and BMI were not relevant to SJL, confirming the classical correlation analysis and *t* test results mentioned above.

We next used classical statistics to explore the relationship between SJL and age/nocturnal sleep/daytime naps. Although the *P* values suggested highly significant results (*P*<.001), the relatively small correlation coefficients suggested that SJL did not correlate with age in all subjects (*r*=-0.03, *P*<.001). Linear regression analysis was used to quantify the relationship between SJL and daytime naps. The fitted slope of nap durations was 0.0066 and *P*<.001 (ie, if the daytime nap increased 1 minute, the SJL approximately increased 0.0066*60=0.4 minutes). When adding nocturnal sleep duration into the regression model, the fitted slope of daytime nap was not significant any more (*P*=.16) but nocturnal sleep duration became significant (the fitted slope was 0.0007 and *P*<.001). These results suggested a positive relationship between SJL and durations of daytime nap/nocturnal sleep, indicating that people who need more sleep seemed to have larger SJL.

## Discussion

### Principal Considerations

This is the first study characterizing sleep duration, chronotype (ie, mid-sleep points on free days), and social jetlag in a large Chinese population with objective data assessed by wearable devices. Our results reveal that the majority of Chinese people take daytime naps (66,953/71,176, 94.07%), and most importantly only a minor proportion (12,151/71,176, 17.07%) of the Chinese population have SJL longer than 1 hour, which is much smaller than the 69% reported in a European population [5]. Remarkably, in contrast to the results shown in previous studies that SJL is associated with obesity [5,33,34], we report that SJL does not associate with BMI in a Chinese population. Surprisingly, a high proportion of Chinese people, that is, 31.53% (22,442/71,176) of all the subjects or 31.16% (15,447/49,573) of the subjects whose chronotypes are available, have negative SJL. Among these people, the majorities are early (7127/15,447, 46.14%) and intermediate (7396/15,447, 47.88%) chronotypes. In fact, we found that more than half (7127/13,266, 53.72%) of the whole early types show negative SJL, indicating that negative SJL may be a common phenomenon in people of early chronotypes. In contrast to our original hypothesis that daytime naps reduce SJL, we found that people sleeping longer during daytime naps also have a larger SJL. We further showed that chronotype (ie, MSFsc), age, and total sleep duration (ie, the sum of nocturnal and daytime sleep durations) are the most important factors associating with SJL. People of later chronotypes and long sleepers have a larger SJL. In addition, we report a higher proportion of early chronotypes (13,266/49,573, 26.76%) than late chronotypes (7257/49,573, 14.64%) and show the age-MSFsc relationship in Chinese population as previously reported in the Western countries [30,35,36], confirming that MSFsc becomes later during adolescence. However, its peak is earlier than the one reported in the German-speaking countries in 2004 [30] (ie, 4:00 am vs 5:00 am), indicating that nowadays the shift of MSFsc towards later chronotypes during adolescence is smaller compared to 10 years ago. We did not find gender differences in MSFsc.

### Sleep Duration

The average nocturnal sleep duration is less than 7 hours in our subjects, which is shorter than that reported in other countries like the United States [,] and European countries [37]. In fact, at least 7 hours of sleep is recommended by the US Centers for Disease Control and Prevention to promote optimal health [38]. However, the majority of the Chinese population taking daytime naps results in an average total sleep duration of 7 hours (ie, 419 minutes [SD 47 minutes]). Our results also show gender differences in total sleep duration, that is, females sleep on average 17 minutes longer than males, which is consistent with the results of previous studies [39,40].

### Early Chronotypes in the Chinese Population

This is the first study that chronotyped the Chinese population with an objective assessment. Very few previous studies have evaluated the chronotypes of Chinese using subjective questionnaires, and their results have been inconsistent. For example, Li et al [15] and Carciofo et al [16] validated the MEQ questionnaire with a sample of 188 subjects from the Beijing district and a sample of 305 Beijing residents. They found inconsistent results of chronotypes classified with the Horne and Östberg cut-off criteria [41]. Carciofo et al reported that 48.9% of subjects were neutral type, 44.3% were definitely or moderately morning types, and 6.9% definitely or moderately evening types [16]. Li et al showed that 66% of subjects were definitely or moderately morning types, 31% were neutral type, and 3% definitely or moderately evening types [15]. However, both studies indicated a prevalence of early type compared to late chronotypes in China. In our results, the objective MSFsc in the Chinese population follows a near normal distribution (Figure 4) similar to the ones shown in a recent study done in the US with a questionnaire [35]. With the same arbitrary cut-offs used in the German-speaking populations [31] and in the US population [35], we also found a higher proportion of early than late types. Therefore, our results of objective assessment of chronotypes agree with the findings of previous studies in Chinese using subjective questionnaires.

### Age Differences in Chronotypes

Our results (Figure 5) show that Chinese adolescents tend to be later chronotypes than other age groups consistent with previous results reported in other countries such as German-speaking countries [30,36] and the United States [35]. The big advantage of our study is that the MSFsc was calculated from objective measurement of sleep schedules by wearable devices, whereas in previous studies only subjective MSFsc derived from questionnaires was available. Both objective and subjective assessments corroborate a later chronotype in adolescents. The objectively assessed peak of age related to the MSFsc curve is 4:00 am at the age of 22 years in Chinese and is 5:00 am at age 20 years in the German-speaking population assessed by the MCTQ in 2004 [30]. Thus, our results suggest that although currently the MSFsc still shifts towards later chronotypes in adolescents, the degree of the shift may be decreasing compared to 15 years ago or may be smaller when objectively assessed with wearable devices. This hypothesis is supported by data from the United States [35], as the authors compare the MSFsc of their survey years (2003-2014) and find a trend towards earlier chronotypes in the later survey years (2011-2014) compared to the early ones.

The peak value of MSFsc at age 22 years is not significantly different from the ones at the ages 16-26 years. This range of age is wider than the one shown in the German-speaking population (ie, 19-23 years) [30]. The 24-hour sleep-wake cycle is influenced by genetic, molecular, lifestyle, societal (eg, cultures, working hours, occupation), environmental (eg, pollution, climate), geographical, and other factors. Thus, these factors may also influence the calculation of the MSFsc (the chronotypes) [42]. As Roenneberg et al suggested that the peak of age-MSFsc curve is a marker for the end of adolescence [30], the genetic factors may account for the aforementioned differences among different countries. Other factors like societal, environmental, and geographical factors could also influence the age-MSFsc relationships. For example, China covers a larger range of latitude (ie, from the northern tropic to high latitudes) and longitude than the German-speaking countries (eg, Germany and Switzerland are at high latitudes). Higher latitudes are associated with higher eveningness [10]. Therefore, when we average the MSFsc of Chinese people across the whole country, it is plausible that the Chinese have a smaller value but wider age ranges of the latest MSFsc compared to the German-speaking population.

### Gender Differences in Chronotypes

In contrast to the results of previous studies, our objective data do not confirm gender differences in the Chinese population. In fact, to the best of our knowledge, the gender differences in chronotypes are reported inconsistently in the literature. Large sample and meta-analysis studies have found a slight tendency towards early chronotypes in females compared to males [30,35,36,43,44], but some studies report controversial findings [45,46] or no gender differences [43]. For example, a study [45] comparing the Morningness-Eveningness Stability Scale among Iranian, Spanish, and German populations found that the morning affect is higher in Spaniards and German females compared to Spaniards and German males. Iranian females, however, reported lower morning affect scores compared to Iranian males, indicating that gender differences in chronotypes are controversial between Iranian and Spaniard/German populations. In contrast to the results of most studies done in Europe showing gender differences [36,44], Duarte et al reported no significant gender differences in age groups between 30 and 44 years in Brazil using the MEQ questionnaire [47]. In Finland, conflicting results were also reported [46]. Evening type is more common among women than men in the Finnish population.

### Negative Social Jetlag

MSFsc is positively correlated to SJL, corroborating the notion that people of later types may have higher SJL [5] because currently human society in industrialized countries favors early over later working hours. However, our results also report negative SJL in quite a number of Chinese, especially in early types. Negative SJL has been reported in previous studies [5], and Roenneberg et al suggested that this may be because people of early types sleep later than their circadian sleep window during work days, thus on free days they go to sleep earlier [5]. Other possible explanations of the negative SJL include shift workers who work on weekends, or accumulated sleep pressures forcing some people to go to sleep earlier on free days (ie, sleeping in is not the only way to compensate for insufficient sleep; some people also prefer to sleep earlier on free days). Nevertheless, our results report for the first time a relative high prevalence of negative SJL in a larger population (ie, Chinese) with a higher proportion of early chronotypes than late chronotypes. To the best of our knowledge, the health consequences of negative SJL have been rarely investigated in previous studies, probably due to the relative low proportion of early chronotypes/negative SJL in the studied populations (eg, the absolute values of SJL were calculated as very few subjects reported negative SJL in Roenneberg et al [5]). Only one recent study done in Japan shows that about 22.3% of Japanese children and adolescents report negative SJL and the students with negative SJL less than 1 hour have poorer academic performance [48]. Therefore, we suggest that more data are needed to explore the potential influences of negative SJL in human health, especially for early chronotypes whose sleep windows may be delayed during the work days. These data will help us better understand how the misalignments of biological and social time influence human health. Previous studies emphasized the influences of SJL on late chronotypes but ignored its influences on early chronotypes. For example, late chronotypes suffer from earlier waking in the morning, so later school/work start times have been recommended in some countries. But early chronotypes may equally suffer from later sleep onset during the night and their circadian clock prefers an early school/work start. So, reducing the negative SJL should be equally important for public health, especially for early chronotypes.

### Age and Social Jetlag

Age is suggested to be associated with SJL by our random forest model, but it is not confirmed by classical correlation analysis as the correlation coefficient is too small (*r*=-0.03) in spite of small *P* value (*P*<.001). This result could be explained by the nonlinear relationship between age and SJL, similar to the one between age and MSFsc. As shown in our results (Figure 5), children are early chronotypes and become progressively later in adolescence. Chronotypes are positively correlated to SJL, so SJL is increasing with maturation in childhood. In adulthood, MSFsc is decreasing with increased age (ie, chronotypes become earlier again); thus, SJL is decreasing with age. This nonlinear pattern cannot be discovered by linear correlation analysis, but it can be recognized by random forest, a decision tree–based algorithm suitable to discovery of nonlinear relationships between predictors and dependent variable.

### Body Mass Index and Social Jetlag

In contrast to the results of previous studies [5,33,34], we could not find the association between SJL and BMI in the Chinese population. In fact, we are not the only study reporting no association between SJL and BMI. Similar results have been recently reported in Russia [49], Norway [50], and Czech Republic [51]. Thus, societal or geographical (eg, Russia and Norway are of higher latitudes compared to Germany) factors could account for the inconsistent results. Different data analysis methods may also influence the results. For example, both our analyses with machine learning (ie, random forest) and correlation analysis failed to reveal an association between SJL and BMI in our data. However, if we used ordinary least squares regression with BMI, gender, age, exercise, and sleep duration as predictors, we could find BMI as a positive predictor (coefficient is 0.0033, and *P*=.017) for SJL. However, this regression analysis is problematic due to the multicollinearity of covariates. In addition, energy expenditure and energy intake have not been considered in any of these studies including this one. Although we have taken into account the count of daily walking steps measured by wearable devices, which is an indicator of exercise, we have no indicator of energy intake. Therefore, the link between SJL and obesity needs further research.

### Daytime Naps and Social Jetlag

The unexpected positive rather than negative relationship between SJL and daytime naps is best explained by higher sleep need. First, our data show that the total sleep durations on work days and free days are correlated (*r*=0.41, *P*<.001), indicating that those people sleeping longer are long sleepers while the people without daytime naps have shorter total sleep durations and are likely to be short sleepers. Second, our result of an average of 7 hours total sleep duration already indicates that in general Chinese people suffer from insufficient sleep. People taking longer daytime naps sleep less during the night but have longer total sleep time (Figure 3), suggesting that they show a disposition to long sleep need (ie, long sleepers) but suffer from insufficient nocturnal sleep. Therefore, they sleep longer on free days, resulting in larger SJL (ie, longer daytime nap is associated with larger SJL). These results also indicate that long sleepers have larger SJL.

The positive association between daytime naps and SJL should not be considered to reflect a causal relationship and misinterpreted in that a longer daytime nap leads to larger SJL. In contrast, we think that daytime naps are still helpful to reduce SJL. However, they are not long enough to totally compensate for insufficient nocturnal sleep because the majority of Chinese people take daytime naps and have less than 1-hour SJL compared to European populations (ie, assuming that if Chinese people did not take daytime naps, they are likely to have higher sleep debts and thus larger SJL).

We also recognize that long daytime naps may also be associated with other health problems such as diabetes [52] or cardiovascular diseases [53]. Previous studies suggested that long daytime naps (ie, longer than 1 hour) can significantly increase the odds ratio of diabetes mellitus [52,54] and are associated with a higher risk of cardiovascular disease [53], but short naps (less than 30 minutes) are suitable to promote health. The influences/impacts of daytime napping duration on human health are less conclusive and definitely deserve more study. For example, long daytime naps may indicate sleep deprivation or disturbed nocturnal sleep (eg, people with sleep disorders such as sleep apnea, parasomnia, or insomnia). So, the increased risk of cardiovascular disease or diabetes in those people may be due to their poor nocturnal sleep or sleep disorders, rather than long daytime naps.

### Limitations

There are several limitations to our study. First, we are well aware that the cut-offs of MSFsc classifying chronotypes are sensitive to the study population [55] and other factors like latitude and longitude [10]; thus, they should be interpreted with caution. Our results that nearly half of negative SJL are intermediate chronotypes may indicate that the proportion of early chronotypes in this study is underestimated using the cut-offs.

Second, we have no data on the subjective estimations of sleep. To correlate the objective measures and subjective evaluations (eg, sleep schedule, daytime sleepiness) may be an interesting topic in the future. Third, in China the majority of employees can work only under the standard working hour system, which limits them to work 8 hours per day and 40 hours per week. Therefore, most Chinese people work from Monday to Friday. But working on Saturday or Sunday (not on both days as it is forbidden to work for the whole week according to Chinese law) with overtime payment is allowed in China. In this case, the definition of free days in our study needs to be adjusted in a small proportion of subjects. We suggest that future studies should take this into account. For example, the questionnaires used to assess chronotypes and SJL should include working hours/days per week of the subjects. The objective recordings with wearable devices or mobile phones should be able to classify work days and free days. In addition, we are aware that seasonal effects can influence chronotypes [36] and they should be controlled as suggested in previous studies [56]. Therefore, we recorded our subjects from April to July, when the temperature is warm and the duration of daytime sunshine is increasing. It would be interesting in the future to compare our results with the data measured by wearable devices from winter when the temperature is cold and the night is longer. These objective data collected from large populations will help us better understand how daylight and temperature influence human circadian rhythm and health. For example, they can contribute to the ongoing debate of whether we should stop shifting to daylight saving time in summer in Europe and North America and instead use permanent summer time or winter time.

Finally, we have no information of the timing but only the durations of daytime naps in the database. It is reasonable to speculate that the majority of our subjects may regularly take naps after lunch between 12:00 to 14:00 (typical midday break time in China) because noon napping is an intrinsic part of Chinese culture. But we acknowledge that this speculation needs to be tested in future studies. It is an interesting topic to investigate whether the timing or regularity of naps may influence nocturnal sleep or the calculation of MSFsc and SJL.

### Conclusions

Our study characterizes for the first time the chronotypes and social jetlag in a large Chinese population measured using wearable devices. We found a higher proportion of early compared to late chronotypes in Chinese. Chinese had less SJL than the results reported in European populations probably because of their noon napping culture. We also found that people of later chronotypes and long sleepers suffered more from SJL, but larger SJL was not associated with higher BMI. Surprisingly, more than half of the early chronotypes had negative SJL. We suggest that future studies are needed to further investigate the relationships between negative SJL and human health. Our study demonstrates that these days the modern wearable technologies tracking sleep-wake patterns are becoming powerful tools in the field of chronobiology.

## Abbreviations

BMI: body mass index
CPC: cardiopulmonary coupling
MCTQ: Munich ChronoType Questionnaire
MEQ: Morningness-Eveningness Questionnaire
MSFsc: adjusted mid-point of sleep on free days
SJL: social jetlag

## References

[1] Wittmann M, Dinich J, Merrow M, Roenneberg T (2006). Social jetlag: misalignment of biological and social time. Chronobiol Int.

[2] Amaral FG, Castrucci AM, Cipolla-Neto J, Poletini MO, Mendez N, Richter HG, Sellix MT (2014). Environmental control of biological rhythms: effects on development, fertility and metabolism. J Neuroendocrinol.

[3] Obradovich N, Migliorini R, Mednick SC, Fowler JH (2017). Nighttime temperature and human sleep loss in a changing climate. Sci Adv.

[4] Swaminathan K, Klerman EB, Phillips AJK (2017). Are Individual Differences in Sleep and Circadian Timing Amplified by Use of Artificial Light Sources?. J Biol Rhythms.

[5] Roenneberg T, Allebrandt KV, Merrow M, Vetter C (2012). Social jetlag and obesity. Curr Biol.

[6] Komada Y, Breugelmans R, Drake CL, Nakajima S, Tamura N, Tanaka H, Inoue S, Inoue Y (2016). Social jetlag affects subjective daytime sleepiness in school-aged children and adolescents: A study using the Japanese version of the Pediatric Daytime Sleepiness Scale (PDSS-J). Chronobiol Int.

[7] Martin JS, Hébert M, Ledoux E, Gaudreault M, Laberge L (2012). Relationship of chronotype to sleep, light exposure, and work-related fatigue in student workers. Chronobiol Int.

[8] Levandovski R, Dantas G, Fernandes LC, Caumo W, Torres I, Roenneberg T, Hidalgo MPL, Allebrandt KV (2011). Depression scores associate with chronotype and social jetlag in a rural population. Chronobiol Int.

[9] Wong PM, Hasler BP, Kamarck TW, Muldoon MF, Manuck SB (2015). Social Jetlag, Chronotype, and Cardiometabolic Risk. J Clin Endocrinol Metab.

[10] Randler C, Rahafar A (2017). Latitude affects Morningness-Eveningness: evidence for the environment hypothesis based on a systematic review. Sci Rep.

[11] Adan A, Archer SN, Hidalgo MP, Di Milia L, Natale V, Randler C (2012). Circadian typology: a comprehensive review. Chronobiol Int.

[12] Ohayon MM, Priest RG, Zulley J, Smirne S, Paiva T (2002). Prevalence of narcolepsy symptomatology and diagnosis in the European general population. Neurology.

[13] Li J, Cacchione PZ, Hodgson N, Riegel B, Keenan BT, Scharf MT, Richards KC, Gooneratne NS (2017). Afternoon Napping and Cognition in Chinese Older Adults: Findings from the China Health and Retirement Longitudinal Study Baseline Assessment. J Am Geriatr Soc.

[14] Roenneberg T, Wirz-Justice A, Merrow M (2003). Life between clocks: daily temporal patterns of human chronotypes. J Biol Rhythms.

[15] Li S, Li Q, Wang X, Liu L, Liu Y, Zhang L, Zhang B, Lu L (2011). Preliminary test for the Chinese version of the Morningness-Eveningness Questionnaire. Sleep and Biological Rhythms.

[16] Carciofo R, Du F, Song N, Qi Y, Zhang K (2012). Age-related chronotype differences in Chinese, and reliability assessment of a reduced version of the Chinese Morningness-Eveningness Questionnaire. Sleep and Biological Rhythms.

[17] Kushida C, Chang A, Gadkary C, Guilleminault C, Carrillo O, Dement WC (2001). Comparison of actigraphic, polysomnographic, and subjective assessment of sleep parameters in sleep-disordered patients. Sleep Med.

[18] Pollak C, Tryon W, Nagaraja H, Dzwonczyk R (2001). How accurately does wrist actigraphy identify the states of sleep and wakefulness?. Sleep.

[19] Paquet J, Kawinska A, Carrier J (2007). Wake detection capacity of actigraphy during sleep. Sleep.

[20] de Zambotti M, Baker FC, Colrain IM (2015). Validation of Sleep-Tracking Technology Compared with Polysomnography in Adolescents. Sleep.

[21] Poyares D, Hirotsu C, Tufik S (2015). Fitness Tracker to Assess Sleep: Beyond the Market. Sleep.

[22] Fonseca P, Weysen T, Goelema M, Møst EIS, Radha M, Lunsingh Scheurleer C, van den Heuvel L, Aarts RM (2017). Validation of Photoplethysmography-Based Sleep Staging Compared With Polysomnography in Healthy Middle-Aged Adults. Sleep.

[23] Thomas R, Wood C, Bianchi MT (2017). Cardiopulmonary coupling spectrogram as an ambulatory clinical biomarker of sleep stability and quality in health, sleep apnea and insomnia. Sleep.

[24] Reimer U, Emmenegger S, Maier E, Zhang Z (2017). Recognizing Sleep Stages with Wearable Sensors in Everyday Settings.

[25] Thomas R, Mietus J, Peng C, Goldberger AL (2005). An electrocardiogram-based technique to assess cardiopulmonary coupling during sleep. Sleep.

[26] Domingues A, Paiva T, Sanches JM (2014). Hypnogram and sleep parameter computation from activity and cardiovascular data. IEEE Trans Biomed Eng.

[27] Fagherazzi G, El Fatouhi D, Bellicha A, El Gareh A, Affret A, Dow C, Delrieu L, Vegreville M, Normand A, Oppert J, Severi G (2017). An International Study on the Determinants of Poor Sleep Amongst 15,000 Users of Connected Devices. J Med Internet Res.

[28] Wen D, Zhang X, Liu X, Lei J (2017). Evaluating the Consistency of Current Mainstream Wearable Devices in Health Monitoring: A Comparison Under Free-Living Conditions. J Med Internet Res.

[29] Zhang Z, Henzmann S, Hügli G, Qi M, Chen W, Lu C, Khatami R (2018). Validation of Wearable Sleep Monitoring Device Based on Cardiopulmonary Coupling and Accelerometer with Comparison to Polysomnography in Adults. https://tinyurl.com/yyq7aw6n.

[30] Roenneberg T, Kuehnle T, Pramstaller PP, Ricken J, Havel M, Guth A, Merrow M (2004). A marker for the end of adolescence. Curr Biol.

[31] Roenneberg T, Kuehnle T, Juda M, Kantermann T, Allebrandt K, Gordijn M, Merrow M (2007). Epidemiology of the human circadian clock. Sleep Med Rev.

[32] Breiman L (2001). Random Forests. Machine Learning.

[33] Parsons MJ, Moffitt TE, Gregory AM, Goldman-Mellor S, Nolan PM, Poulton R, Caspi A (2015). Social jetlag, obesity and metabolic disorder: investigation in a cohort study. Int J Obes (Lond).

[34] Mota MC, Silva CM, Balieiro LCT, Fahmy WM, Crispim CA (2017). Social jetlag and metabolic control in non-communicable chronic diseases: a study addressing different obesity statuses. Sci Rep.

[35] Fischer D, Lombardi DA, Marucci-Wellman H, Roenneberg T (2017). Chronotypes in the US - Influence of age and sex. PLoS One.

[36] Randler C, Freyth-Weber K, Rahafar A, Florez Jurado A, Kriegs JO (2016). Morningness-eveningness in a large sample of German adolescents and adults. Heliyon.

[37] Zomers M, Hulsegge G, van Oostrom SH, Proper K, Verschuren W, Picavet HJ (2017). Characterizing Adult Sleep Behavior Over 20 Years-The Population-Based Doetinchem Cohort Study. Sleep.

[38] Liu Y, Wheaton A, Chapman D, Cunningham T, Lu H, Croft JB (2016). Prevalence of Healthy Sleep Duration among Adults--United States, 2014. MMWR Morb Mortal Wkly Rep.

[39] Reyner L, Horne J, Reyner A (1995). Gender- and age-related differences in sleep determined by home-recorded sleep logs and actimetry from 400 adults. Sleep.

[40] Goel N, Kim H, Lao RP (2005). Gender differences in polysomnographic sleep in young healthy sleepers. Chronobiol Int.

[41] Horne J, Ostberg O (1976). A self-assessment questionnaire to determine morningness-eveningness in human circadian rhythms. Int J Chronobiol.

[42] Randler C (2008). Morningness-eveningness comparison in adolescents from different countries around the world. Chronobiol Int.

[43] Randler C (2007). Gender differences in morningness–eveningness assessed by self-report questionnaires: A meta-analysis. Personality and Individual Differences.

[44] Tonetti L, Fabbri M, Natale V (2008). Sex difference in sleep-time preference and sleep need: a cross-sectional survey among Italian pre-adolescents, adolescents, and adults. Chronobiol Int.

[45] Rahafar A, Randler C, Díaz-Morales JF, Kasaeian A, Heidari Z (2017). Cross-cultural validity of Morningness-Eveningness Stability Scale improved (MESSi) in Iran, Spain and Germany. Chronobiol Int.

[46] Merikanto I, Kronholm E, Peltonen M, Laatikainen T, Lahti T, Partonen T (2012). Relation of chronotype to sleep complaints in the general Finnish population. Chronobiol Int.

[47] Duarte L, Menna-Barreto L, Miguel M, Louzada F, Araújo J, Alam M, Areas R, Pedrazzoli M (2014). Chronotype ontogeny related to gender. Braz J Med Biol Res.

[48] Kohyama J (2017). Self-Reported Academic Performance and Lifestyle Habits of School Children in Japan. Int J Child Health Nutr.

[49] Polugrudov AS, Panev AS, Smirnov VV, Paderin NM, Borisenkov MF, Popov SV (2016). Wrist temperature and cortisol awakening response in humans with social jetlag in the North. Chronobiol Int.

[50] Johnsen MT, Wynn R, Bratlid T (2013). Optimal sleep duration in the subarctic with respect to obesity risk is 8-9 hours. PLoS One.

[51] Farkova E, Mankova D, Koprivova J (2017). Is increased body mass index associated with larger social jet-lag?. Sleep Medicine.

[52] Yamada T, Shojima N, Yamauchi T, Kadowaki T (2016). J-curve relation between daytime nap duration and type 2 diabetes or metabolic syndrome: A dose-response meta-analysis. Sci Rep.

[53] Yamada T, Hara K, Shojima N, Yamauchi T, Kadowaki T (2015). Daytime Napping and the Risk of Cardiovascular Disease and All-Cause Mortality: A Prospective Study and Dose-Response Meta-Analysis. Sleep.

[54] Guo VY, Cao B, Wong CKH, Yu EYT (2017). The association between daytime napping and risk of diabetes: a systematic review and meta-analysis of observational studies. Sleep Med.

[55] Malone SK, Patterson F, Lu Y, Lozano A, Hanlon A (2016). Ethnic differences in sleep duration and morning-evening type in a population sample. Chronobiol Int.

[56] Randler C, Prokop P, Sahu S, Haldar P (2015). Cross-cultural comparison of seven morningness and sleep-wake measures from Germany, India and Slovakia. Int J Psychol.

